# Intestinal nonrotation with left-sided appendiceal microperforation mimicking colonic perforation: CT diagnostic pitfall and successful conservative treatment

**DOI:** 10.1016/j.radcr.2026.08.032

**Published:** 2026-09-07

**Authors:** Wen-Xi Han, Lang Wang, Jun-Lin Huang, Jun-Ming Li, Pei-Lun Han, Cheng-Long Wang

**Affiliations:** a Department of Radiology, West China School of Public Health and West China Fourth Hospital, Sichuan University, Chengdu, China; b School of Nursing, Anhui University of Chinese Medicine, AnHui, China

**Keywords:** Intestinal nonrotation, Left-sided appendicitis, Appendiceal microperforation, Left lower quadrant pain, Diagnostic pitfall

## Abstract

Intestinal nonrotation is an uncommon subtype of intestinal malrotation that may place the cecum and appendix in the left lower quadrant, creating a diagnostic pitfall in patients with left-sided abdominal pain. A 49-year-old man presented with a 3-day history of progressive left lower quadrant pain, fever, vomiting, and diarrhea. Contrast-enhanced CT demonstrated predominantly right-sided small bowel and left-sided colon, with the cecum and appendix located in the left iliac fossa. The appendix measured 9 mm in diameter and showed wall thickening, hyperenhancement, surrounding fat stranding, a suspected focal wall discontinuity, and adjacent tiny extraluminal air foci, findings strongly suggestive of appendiceal microperforation. The neighboring sigmoid colon showed mild focal wall thickening and fat stranding, a small localized abscess was also identified adjacent to the sigmoid colon, but no definite colonic wall defect, or inflamed diverticulum. After CT re-evaluation and surgical consultation, the initial concern for sigmoid perforation was revised to left-sided appendicitis with CT-suspected microperforation. Given the localized imaging findings and clinical stability, the patient was managed nonoperatively with antibiotics and close monitoring, with subsequent clinical and radiologic improvement. This case emphasizes the importance of identifying the malpositioned cecum and appendix, determining the inflammatory epicenter, and assessing the distribution of extraluminal air in patients with left lower quadrant pain.

## Introduction

Intestinal nonrotation is a subtype of intestinal malrotation resulting from failure of normal embryologic midgut rotation. In this anatomical pattern, the small bowel is located predominantly in the right abdomen, whereas the colon is located predominantly in the left abdomen, and the cecum may be positioned in the left lower quadrant [1]. The reported prevalence in adults based on CT colonography is approximately 0.2% [1]. Acute appendicitis occurring in patients with intestinal nonrotation is rare and poses a significant diagnostic challenge due to atypical pain localization (left lower quadrant) and imaging findings that can mimic more common conditions such as colonic diverticulitis or perforation [2].

While several case reports describe left-sided appendicitis in the setting of intestinal malrotation, most focus on surgical management of overt appendicitis or abscess formation [3,4]. There is a paucity of literature detailing the specific CT features that differentiate appendiceal microperforation from primary colonic perforation in these patients, a distinction critical for guiding appropriate management. Furthermore, successful nonoperative management of appendiceal microperforation in this anatomical context is scarcely reported.

This case report aims to: (1) Describe the detailed CT findings of intestinal nonrotation with left-sided appendiceal microperforation mimicking colonic perforation; (2) Systematically analyze the imaging pitfalls and key differentiating features; (3) Summarize the diagnostic challenges reported in the literature for similar conditions; and (4) Report the successful outcome of conservative antibiotic therapy, providing a management alternative to immediate surgery.

## Case report

A previously healthy 49-year-old male presented to the emergency department with a 3-day history of progressive, constant pain in the left lower quadrant. The pain was associated with local tenderness, rebound tenderness, vomiting, diarrhea, and fever (39°C). He denied any history of similar episodes, abdominal surgery, or known congenital anomalies. Physical examination revealed localized guarding and rebound tenderness in the left lower abdomen, without diffuse abdominal rigidity. The patient was hemodynamically stable at presentation. Laboratory tests showed an elevated white blood cell count of 10.01 × 10⁹/L (normal range: 4.0–9.5 × 10⁹/L) with neutrophilia (87%).

### Imaging findings

**Fig. 1:**
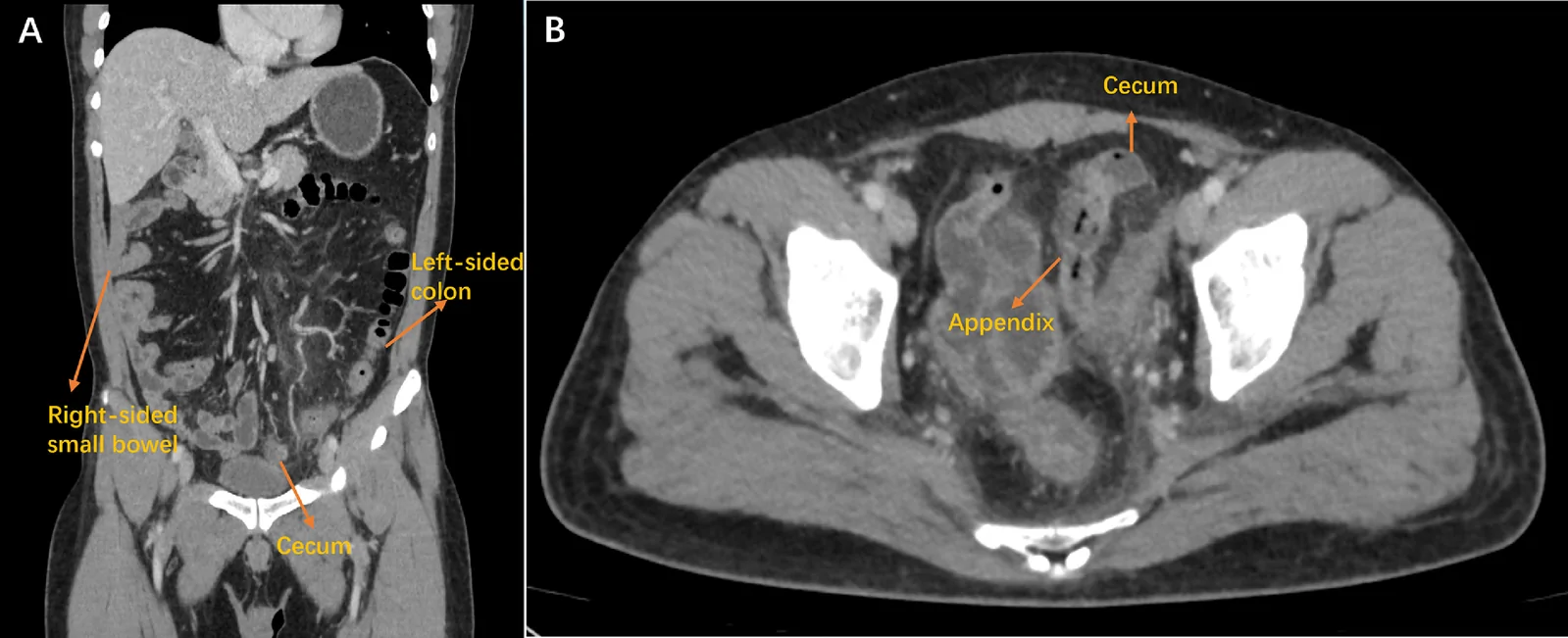
CT demonstration of intestinal nonrotation and the left-sided appendix. (A) Coronal multiplanar reconstruction demonstrates predominantly right-sided small bowel and left-sided colon, with the cecum located in the left lower abdomen. (B) Axial image demonstrates the appendiceal base arising from the malpositioned cecum, and the course of the blind-ending appendix in the left lower quadrant.

Contrast-enhanced abdominal CT demonstrated predominantly right-sided small bowel and left-sided colon, consistent with intestinal nonrotation. The cecum was located in the left iliac fossa. On axial and coronal multiplanar reconstructions, the appendix was traced from the malpositioned cecum as a blind-ending tubular structure. It measured 9 mm in maximal outer diameter and demonstrated circumferential wall thickening, mural hyperenhancement, and surrounding fat stranding (Fig. 1).

**Fig. 2:**
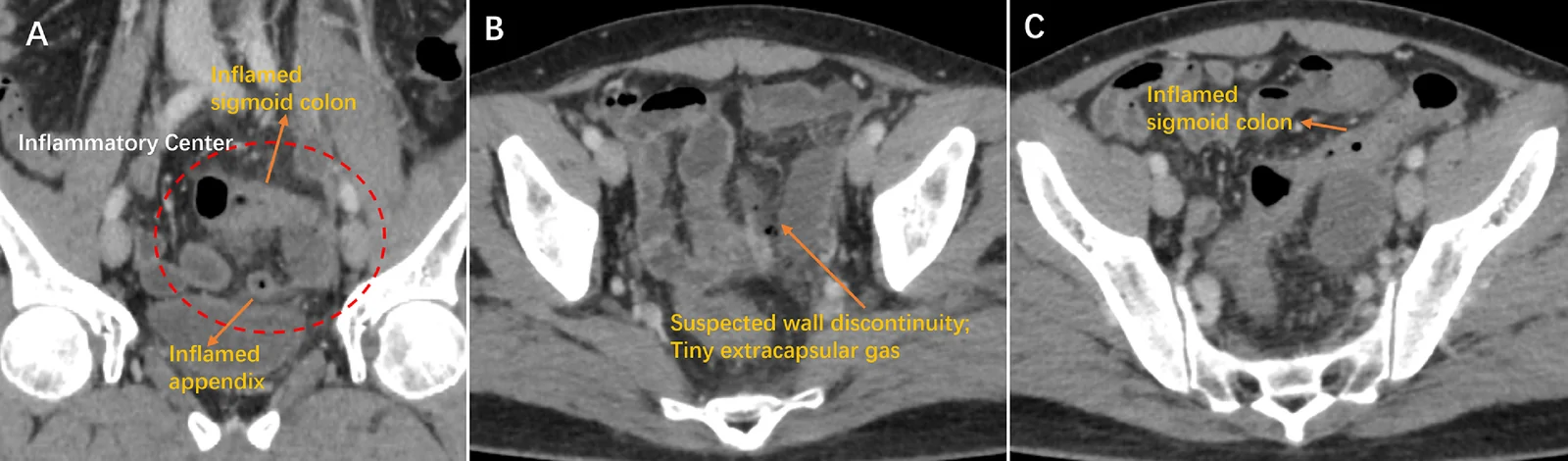
CT findings favoring left-sided appendicitis with suspected microperforation over primary sigmoid perforation. (A) Axial contrast-enhanced CT image demonstrates a dilated, thick-walled appendix with surrounding inflammatory change adjacent to the sigmoid colon. (B) Axial image demonstrates a suspected focal discontinuity of the appendiceal wall and tiny extraluminal air foci immediately adjacent to the suspected appendiceal wall discontinuity. (C) The adjacent sigmoid colon shows mild focal wall thickening without a definite wall defect or inflamed diverticulum, favoring secondary reactive change.

A suspected focal discontinuity was identified in the mid appendiceal wall on axial images. Several tiny extraluminal air foci, each measuring less than 2 mm, were located immediately adjacent to the suspected appendiceal wall defect. These findings were considered strongly suggestive of appendiceal microperforation. A small abscess was also found, located adjacent to the sigmoid colon (Fig. 2).

The adjacent sigmoid colon demonstrated mild focal wall thickening and pericolic fat stranding. No definite sigmoid wall defect, inflamed diverticulum, sigmoid-centered inflammatory mass, or bowel ischemia was identified. The overall distribution of inflammation favored an appendiceal source with secondary reactive sigmoid change, although a small contained sigmoid perforation could not be excluded solely on the basis of the subtle local findings.

### Diagnosis and management

Based on the left lower quadrant peritoneal signs and localized extraluminal air adjacent to the sigmoid colon, the initial clinical and CT interpretation favored a contained sigmoid perforation, and surgical consultation was obtained. The CT images were subsequently re-evaluated by an experienced radiologist, who recognized the intestinal nonrotation and traced the inflamed appendix from the left-sided cecum. The inflammatory changes and tiny extraluminal air foci were considered more closely related to the appendix than to the sigmoid colon. The leading diagnosis was therefore revised to left-sided acute appendicitis with CT-suspected microperforation and secondary reactive sigmoid changes.

Although localized guarding and rebound tenderness were present, the patient remained hemodynamically stable and showed no clinical evidence of generalized peritonitis. CT demonstrated only localized extraluminal air, without a large abscess, diffuse pneumoperitoneum, bowel ischemia, or large-volume ascites. After surgical consultation and clinicoradiologic reassessment, immediate operative exploration was deferred in favor of closely monitored nonoperative management. The patient received intravenous cefoperazone sodium and sulbactam sodium at 3 g every 12 hours. Surgical intervention was to be reconsidered if abdominal findings, fever, inflammatory markers, or hemodynamic status worsened, or if an abscess developed. His abdominal pain and fever gradually improved over the following 5 days, and repeat laboratory testing on day 11 demonstrated normalization of the white blood cell count.

### Follow-up

**Fig. 3:**
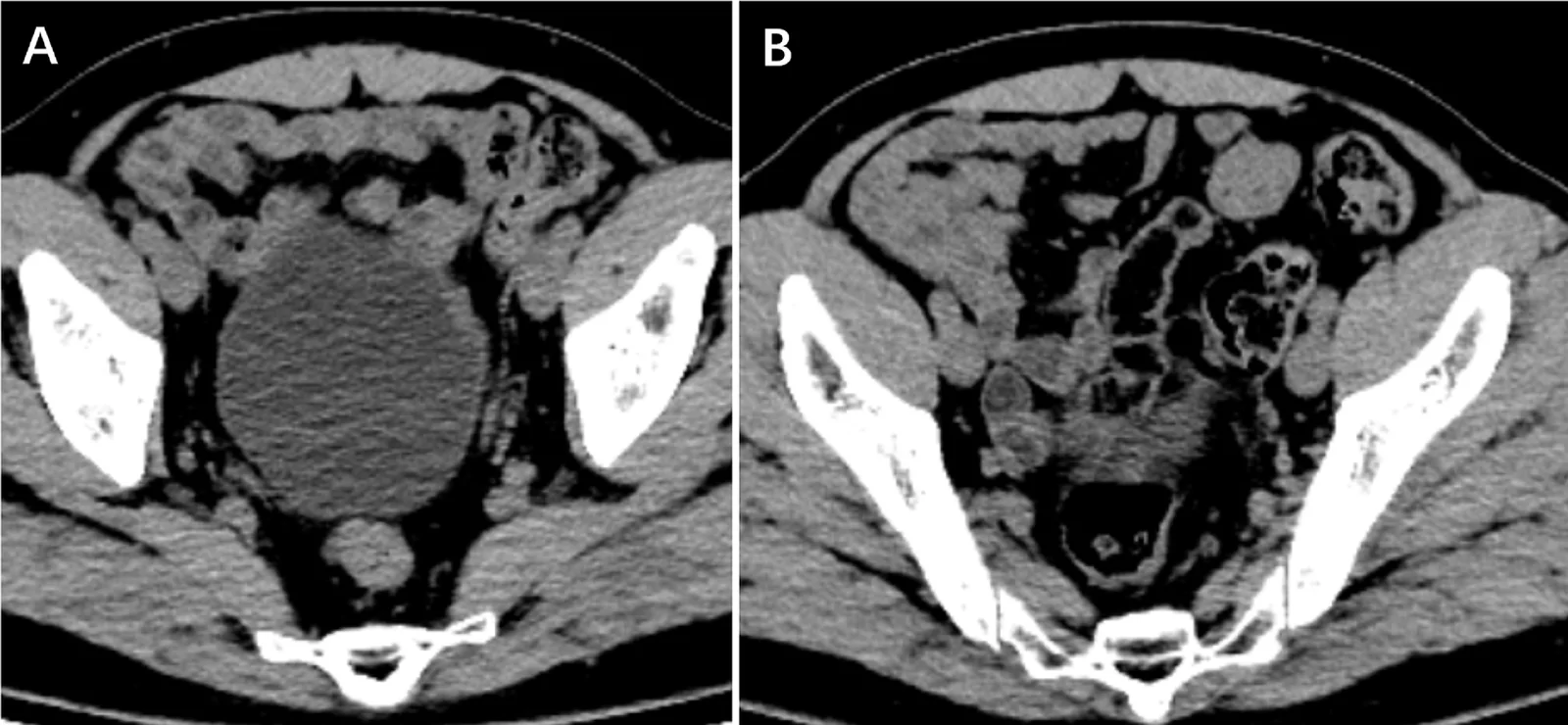
Follow-up CT after non-operative management. Follow-up CT obtained 3 months later at a comparable level demonstrates resolution of the extraluminal air and substantial improvement in the appendiceal and adjacent sigmoid inflammatory changes.

A follow-up pelvic CT scan performed 8 days after the initial presentation demonstrated complete resolution of the previously seen free air foci. There was also marked improvement in the appendiceal wall thickening and periappendiceal fat stranding. The reactive inflammatory changes in the adjacent sigmoid colon had substantially subsided. At the 3-month clinical and CT follow-up, the patient remained asymptomatic. CT showed no apparent residual inflammatory abnormality involving the appendix or adjacent sigmoid colon (Fig. 3).

## Discussion

Intestinal nonrotation is a subtype of intestinal malrotation in which the small bowel is located predominantly in the right abdomen and the colon predominantly in the left abdomen [1]. The cecum and appendix may therefore lie in the left lower quadrant, causing acute appendicitis to present with left-sided abdominal pain and mimic more common left-sided colonic disorders, particularly sigmoid diverticulitis or perforation [2,4,5]. In the present case, the initial concern for sigmoid perforation was understandable because the patient presented with left lower quadrant pain and localized peritoneal irritation, while CT demonstrated extraluminal air and inflammatory changes adjacent to the sigmoid colon. Recognition of the left-sided cecum and direct tracing of the appendix from the cecal base were therefore essential steps in reconsidering the source of inflammation.

Several CT findings may assist in distinguishing appendiceal inflammation or perforation from primary sigmoid disease. An appendiceal source is favored when a dilated and inflamed blind-ending tubular structure can be traced from the cecum, the inflammatory changes are greatest around the appendix, and extraluminal air is clustered adjacent to a suspected appendiceal wall defect [3,6]. In contrast, primary sigmoid diverticulitis or perforation is more strongly suggested by an inflamed diverticulum, a definite colonic wall defect, sigmoid-centered inflammation, a pericolic abscess, or extraluminal air tracking along the sigmoid mesocolon. In the present patient, the appendix was dilated and demonstrated mural thickening, hyperenhancement, surrounding fat stranding, and a suspected focal wall discontinuity. The adjacent sigmoid colon showed mild focal wall thickening and pericolic inflammatory change, but no definite sigmoid wall defect, inflamed diverticulum, or pericolic abscess was identified. These findings favored an appendiceal source with secondary reactive sigmoid involvement.

Nevertheless, no single CT feature can definitively establish the source of a small contained perforation. This distinction is particularly difficult when the inflamed appendix and sigmoid colon are closely apposed. In the present case, the tiny extraluminal air foci were located, immediately adjacent to the suspected appendiceal wall discontinuity and did not track along the sigmoid mesocolon. Therefore, although the combined anatomical and inflammatory findings favored appendiceal microperforation, a small contained sigmoid perforation could not be excluded with absolute certainty on the basis of the subtle local findings alone. Because no surgical or pathological confirmation was available, the diagnosis should be regarded as a clinicoradiologic diagnosis of CT-suspected appendiceal microperforation rather than an anatomically proven perforation.

Appendectomy remains the conventional treatment for perforated appendicitis. However, the management decision in the present case was influenced by both the localized CT findings and the patient’s clinical stability. Although localized guarding and rebound tenderness were present, there was no diffuse abdominal rigidity or other evidence of generalized peritonitis. CT demonstrated only localized extraluminal air, without a large abscess, diffuse pneumoperitoneum, bowel ischemia, or large-volume ascites. Following surgical consultation and clinicoradiologic reassessment, immediate operative exploration was deferred in favor of antibiotics and close clinical and laboratory monitoring. The patient showed progressive symptomatic improvement, normalization of the white blood cell count, and resolution of the extraluminal air and inflammatory changes on follow-up CT. These findings supported the initial nonoperative strategy in this individual patient.

The principal educational value of this case lies not solely in the rarity of left-sided appendicitis in intestinal nonrotation, but in its specific CT mimicry of sigmoid perforation. Systematic reassessment of the bowel anatomy, identification of the appendix from the malpositioned cecum, evaluation of the inflammatory epicenter, and analysis of the extraluminal air distribution changed the leading diagnosis and allowed immediate exploratory surgery to be deferred. However, this single case does not establish the general safety or efficacy of nonoperative treatment for appendiceal microperforation in intestinal nonrotation. Additional limitations include the absence of surgical and pathological confirmation and the relatively short follow-up period, which limits assessment of long-term recurrence risk. Nonoperative management should therefore be considered only in carefully selected, clinically stable patients when close surgical, clinical, laboratory, and imaging surveillance is available.

## Ethical approval

This single-patient, retrospective case report was conducted in accordance with the ethical standards of the institutional and/or national research committee and with the 1964 Helsinki Declaration and its later amendments or comparable ethical standards. Formal approval and waiver of informed consent were obtained from the Institutional Review Board (IRB)/Ethics Committee of West China School of Public Health and West China Fourth Hospital, Sichuan University. The committee granted a waiver for the requirement of obtaining individual patient informed consent specifically for this publication, as the study is retrospective in nature, involves minimal risk, and all patient-identifiable information (including name, ID number, and specific dates) has been completely removed and anonymized from the manuscript and images. The scientific and educational value of reporting this case was deemed to outweigh the need for direct consent.

## Patient consent

Written informed consent was obtained from the patient for the publication of this case report, encompassing all clinical details and accompanying images.
